# Proteolytic Biosensors with Functional Nanomaterials: Current Approaches and Future Challenges

**DOI:** 10.3390/bios13020171

**Published:** 2023-01-21

**Authors:** Jin-Ha Choi

**Affiliations:** School of Chemical Engineering, Clean Energy Research Center, Jeonbuk National University, Jeonju 54896, Republic of Korea; jhchoi@jbnu.ac.kr; Tel.: +82-63-270-4854

**Keywords:** proteolytic biosensors, enzymatic biosensors, matrix metalloproteinase (MMP), caspase family, protease

## Abstract

Proteolytic enzymes are one of the important biomarkers that enable the early diagnosis of several diseases, such as cancers. A specific proteolytic enzyme selectively degrades a certain sequence of a polypeptide. Therefore, a particular proteolytic enzyme can be selectively quantified by changing detectable signals causing degradation of the peptide chain. In addition, by combining polypeptides with various functional nanomaterials, proteolytic enzymes can be measured more sensitively and rapidly. In this paper, proteolytic enzymes that can be measured using a polypeptide degradation method are reviewed and recently studied functional nanomaterials-based proteolytic biosensors are discussed. We anticipate that the proteolytic nanobiosensors addressed in this review will provide valuable information on physiological changes from a cellular level for individual and early diagnosis.

## 1. Introduction

Accurate and early diagnosis is essential for various diseases to reduce the fatality rate and increase the recovery rate [1,2,3]. To this end, there is an urgent need for biosensors that precisely and sensitively measure potential biomarkers such as proteins or nucleic acids. In particular, high-performance biosensors have been actively developed to effectively treat infectious diseases such as SARS-CoV-2 and to help prevent transmission [4,5,6,7,8,9,10]. Among various biomarkers, protein could be used as an accurate diagnosis biomarker since proteins are end products produced by the central dogma process, unlike nucleic acid biomarkers, which have inaccuracies due to post-transcriptional and post-translational processes [11,12]. In general, sandwich immunoassay is the most typical protein detection method using an antigen–antibody binding reaction [13,14,15,16,17]. This immunoassay-based measurement method, represented by enzyme-linked immunosorbent assays (ELISAs), has the advantage of being able to selectively detect various proteins, and the results can be intuitively viewed because the results can be confirmed with the naked eye using an enzyme-substrate reaction. However, at least two antibodies are required to measure the target protein, and colorimetric reactions with labeling enzymes are required to confirm the signal. Due to these disadvantages, there are also severe limitations in that it is cost-ineffective, labor-intensive, and time-consuming. In addition, the detection accuracy of the biosensor could be reduced owing to the complicated process.

Among numerous protein biomarkers, there are many enzymatic proteins, including matrix metalloproteinase (MMP) and the caspase family, with proteolytic properties [18,19,20,21,22,23]. Some of the proteolytic enzymes have the particular property of recognizing and degrading specific peptide sequences. Using this property, many proteolytic biosensors have been developed without using an antigen–antibody reaction [24,25,26]. Compared to immunoassay-based biosensors, proteolytic biosensors have numerous advantages. For example, since the proteolytic biomarker is measured by a specific peptide degradation reaction, there is an advantage in the reduced cost compared to using multi-antibodies, and the measurement time is shortened because of the simple peptide-cleavage reaction step. In addition, since the peptide degradation reaction is composed of just one step, it is easy to induce a reaction at the intracellular level and in vivo system. In particular, developing nanobiosensors using multifunctional nanomaterials has also been actively studied [27,28,29,30,31]. Nanomaterials have a wide surface area, high reaction rate, and ease of immobilizing various biological materials. Therefore, they have a wide range of applications in the biomedical field, including biosensors. In addition, it is possible to simply check the diagnosis results by improving the measurement sensitivity by using various characteristics (optical, electrical, and mechanical properties, etc.) of nanomaterials. In this review, we will investigate proteolytic biosensors integrated with recently announced nanotechnology. In addition, we will discuss the prospects and complementary points of proteolytic biosensors in the field of disease diagnosis.

## 2. Proteolytic Enzymes

### 2.1. Serine Proteases

Nearly one-third of all proteases can be classified as serine proteases. Serine proteases are a large family of protein-degrade enzymes that play a crucial role in processes such as apoptosis, blood coagulation, and inflammation [32,33,34]. Serine proteases are broadly dispersed in nature and found in all cellular metabolisms. Proteases can be grouped into four major clans, which are the SB clan (subtilisin), the PA clan (chymotrypsin), and SF and SC clans that contain various proteases. Approximately 75% of human serine proteases are part of the PA clan. The PA serine proteases can be divided into three subgroups: the trypsin-like serine proteases, which cleave peptide substrates after positively charged arginine and lysine residues; chymotrypsin-like serine proteases, which cleave after large hydrophobic amino acids, including leucine and alanine; elastase-like enzymes, which cleave substrates after hydrophobic residues. In particular, the neutrophil elastase family, which is related to the immune response, is part of this subgroup [35]. This serine protease family plays an important role in the control of intracellular and extracellular activities. For example, several serine proteases are collected inside granules attached to proteoglycans, avoiding leakage into the cytoplasm and communicating with their cellular objectives [36]. On the other hand, serpins, which were first recognized for serine protease inhibition, inhibit their target protease via a unique suicide mechanism, blocking the protease into an irreversible state [37]. Generally, serine proteases are endoproteases that degrade polypeptide bonds by hydrolysis within a polypeptide chain.

### 2.2. Cysteine Proteases

Cysteine proteases (cathepsins) have been known to be involved in many physiological processes, such as regulation of proteolytic cascades, cytokine maturation, expression of proteins, and antigen presentation from the cell surface [38,39,40]. In addition, cysteine proteases have been understood to be involved in numerous pathologies for a long time [41,42,43]. Cysteine proteases are synthesized as preproenzymes and produced as lysosomes, where they provide their function of protein hydrolysis. After the removal of signal peptides, the molecular mass of these proteases is within the range of 20–35 kDa. Typically, they are involved in precursor protein activation, such as proenzymes and prohormones, bone remodeling, MHC-II-mediated antigen presentation, keratinocytes differentiation, and cell reproduction and apoptosis. In the case of the proteolytic mechanism, Cys25 and His159 form the active site for full catalytic activity. The imidazole group of the histidine polarizes the SH group of the cysteine and enables deprotonation, and a thiolate–imidazolium ion pair is produced. The thiolate anion attacks the carbonyl carbon of the peptide bond to be cleaved, and a tetrahedral intermediate is produced. These cysteine proteases are closely related to several fetal diseases. For example, cathepsin K could be a major target in bone syndromes such as osteoporosis [44,45]. In addition, lots of cancers and arthritis correlate to the expression and activation of cysteine cathepsins [46,47]. Furthermore, the inhibitors of cysteine proteases are highly effective and specific molecules that inhibit cancer progression with fewer adverse effects [48].

### 2.3. Matrix Metalloproteinase (MMP) Family

Matrix metalloproteases (MMPs) are a zinc-dependent endopeptidases family that has a similar structure and the capability to decompose every part of the extracellular matrix (ECM) [49,50,51]. The MMP enzyme essentially consists of extracellular matrix remodeling proteases. Using this important physiological role, they have extensive proteolytic activity and contribute to diverse physiological and pathological processes, such as cancer, chronic wounds, cardiovascular diseases, chronic inflammation, and others. MMPs are universal multi-domain enzymes, with 23 MMPs identified in humans (MMPs 1–3, 7–17, 19–21, 23–28) [52,53,54,55]. The structure of MMPs composes of a propeptide that keeps the inactive state (pro-MMPs) by blocking the interaction between the active site and the substrate by a cysteine switch. The catalytic domain (active site) is characterized by the existence of a Zn-binding site, where the Zn ion is organized by three histidines and a glutamate, resulting in a linker peptide of varying lengths and a hemopexin-like domain. Besides the extracellular matrix (ECM), MMPs are engaged in a number of inter- and intracellular activities and contribute to functional networks, systematically cooperating with other biomolecules. In addition, MMPs are implicated in specific pathological processes; this could lead to the significant application of MMPs as potential biomarkers for the prognosis of disease and early diagnostic approaches with detailed and useful information for effective therapy.

## 3. Extracellular Detection of Protease for Diagnosis Using Nanotechnology

### 3.1. Fluorescence-Based Detection

**Figure 1:**
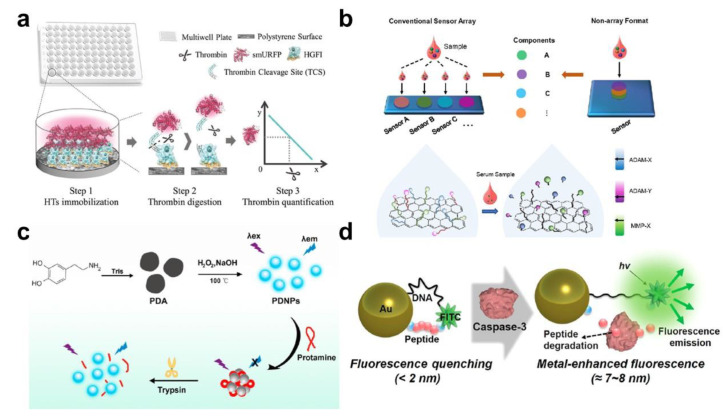
Fluorescence-based biosensors for extracellular protease detection. (**a**) Schematic diagram of the multi-well-plate-based thrombin biosensors composed of trifunctional protein, including a far-red fluorescent protein smURFP, hydrophobin HGFI, and a thrombin cleavage site. This figure is adapted with permission from Ref. [57] (© 2020 WILEY-VCH). (**b**) Schematic diagram of the graphene oxide-based multiplexing biosensor for multiple proteases, ADAM-10, ADAM-17, and MMP-9. This figure is adapted with permission from Ref. [61] (© 2020 American Chemical Society). (**c**) Fluorescent polydopamine nanoparticles for a sensitive and simple trypsin assay via the hydrolysis of protamine by trypsin. This figure is adapted with permission from Ref. [62] (© 2021 Elsevier B.V.). (**d**) MEF-based DNA detection system using a plasmonic Au-assisted MEF effect by CRISPR-Cas12a reaction. This figure is adapted with permission from Ref. [66] (© 2021 American Chemical Society).

Proteases released outside the cell can provide important signals that inform the detailed status of the cell. Thus, extracellular proteases could be one of the important biomarkers for disease diagnosis, and it is necessary to measure them precisely and rapidly for effective diagnosis and therapy. Fluorescence-based biosensors using fluorescent materials, including organic dye and fluorescent nanoparticles, have been widely used to measure extracellular proteases by using the phenomenon in which fluorescence signals are converted by specific reactions between proteases and substrates. Renault et al. showed a novel protease-sensitive fluorescent probe based on the covalent-assembly approach [56]. The protease-sensitive fluorescent probe was designed to be degraded by the targeting enzyme (penicillin G acylase (PGA)) to develop a detectable pyronin fluorescent molecule under physiological conditions through the in situ structure of unsymmetrical pyronin AR116. In addition, c-nucleophile (C-Nu) attached to the pyronin precursor can be screened for the optimization of fluorescent signal generation for sensitive protease measurement. Zhang et al. developed an ultrasensitive and rapid thrombin biosensor composed of trifunctional protein (Figure 1a) [57]. This particular trifunctional protein consists of three functional parts: a thrombin cleavage site (TCS), far-red fluorescent protein (smURFP), and hydrophobin (HGFI). HGFI plays a role in attaching the multi-well plate of the trifunctional proteins, and the TCS is a bridge between the plate and the fluorophore. Once the thrombin cleaves the TCS, a red fluorescent signal can be measured with proportional signal intensity to the thrombin concentration. The detection range of this proteolytic biosensor is from 1.02 aM to 0.01 mM, and the limit of detection (LOD) is 0.2 aM within 20 min. Since the above two proteolytic sensors measure biomarkers based on fluorescent emission, the detection results can be known easily and simply, and the sensitivity is also excellent (aM level). However, there is a limitation in that the measured biomarkers must have proteolytic properties.

For proteolytic detection, fluorescence resonance energy transfer (FRET) is a representative analytical method, which is a distance-dependent fluorescent energy-transfer phenomenon between the donor and the acceptor [58]. If the distance between the donor and acceptor is below 10 nm, the FRET will show and the signal from the acceptor is released, whereas the donor fluorophore is recovered if the distance is above 10 nm. Therefore, distance is a major factor in regulating the FRET phenomenon. In this FRET system, the cleavable peptide sequence, which is a degradable site by a specific protease, can be an essential part of determining the distance between the donor and the acceptor. Zhang et al. exhibited graphene oxide (GO)-assisted fluorescence biosensors for the detection of the human immunodeficiency virus (HIV) protease [59]. Fluorescent-labeled peptide molecules are covalently attached to the surface of GO. Fluorescein is effectively quenched by the FRET effect on GO. Once the HIV protease cleaves the peptide substrate, the fluorescence signal is recovered, proportional to the protease level. The authors claimed that HIV-1 protease could be measured at as low as 1.18 ng/mL in a rapid and accurate manner. Brown et al. revealed a FRET-based biosensor for the detection of the 3-chymotrypsin-like cysteine protease, which is highly related to SARS-CoV-2 [60]. For the FRET effect, the authors used an eCFP (Em: 434 nm) and Venus (Em: 528 nm) pair, connected to the cleavable peptide by a cysteine protease. In addition, it was applied to the high-throughput screening platform for several new inhibitors of SARS-CoV-2. The screening recognized 65 inhibitors, with 20 most active inhibitions of SARS-CoV-2. The aforementioned protease biosensors successfully measured the virus-related protease for the diagnosis of viral infection by using the FRET effect in a simple and highly sensitive manner. FRET-based biosensors have the advantage of measuring target proteases simply and sensitively because they induce changes in fluorescence signals with a single peptide degradation reaction. On the other hand, in the case of virus diagnosis, since nucleic acid biosensors can show relatively accurate diagnostic results for viral diseases, protease biosensors are not yet commonly used diagnostic methods.

Except for virus-related protease detection, other particular proteolytic enzymes, including the MMP family and trypsin, have been measured by integrating the FRET phenomenon. Zhang et al. developed a multiplex fluorescence biosensor for the simultaneous detection of multiple protease activities, such as MMPs and a disintegrin and metalloproteinases (ADAMs) (Figure 1b) [61]. Conventional multiplex biosensing platforms consist of an individual array of different biomarkers, whereas the developed multiplexed nGO−peptide sequence biosensor was fabricated by multiple fluorophore-labeled peptides on an nGO sheet. Using this platform, they found the specific combinations of biomarkers for cancer diagnosis (MMP-9, ADAM-10, and ADAM-17) with joint entropy and programming. Li et al. displayed a sensitive and simple trypsin assay by using stable fluorescent polydopamine nanoparticles with the integration of protamine, which induced the quenching effect (Figure 1c) [62]. Due to the proteolytic effect of trypsin on protamine, the aggregated polydopamine nanoparticle and protamine were degraded, and the fluorescent signal was recovered. An increase in the fluorescent signal was exhibited with the concentration of trypsin (0.01 to 0.1 mg/mL). In addition, this biosensing system showed good practicability in human serum and trypsin inhibitor screening. Xu et al. developed a FRET-based turn-on fluorescent biosensor for trypsin detection based on carbon dots as a donor and Au nanoparticles (AuNPs) as an acceptor [63]. Compared with traditional quantum dots (QDs) and fluorophores, carbon dots have advantages for biosensing applications, such as high photostability, water solubility, and low toxicity. Trypsin-specific peptide sequences (Arg-Cys-Phe-Arg-Gly-Gly-Asp-Asp, RCFRGGDD) were covered AuNPs via the Au-SH bond. Due to the negatively charged ASP-covered AuNPs, the AuNPs were dispersed, and the carbon dot could not emit the fluorescent signal. In the trypsin-degraded peptide sequence, the AuNPs were aggregated, and the carbon dots could recover their fluorescent emission. This system could measure trypsin as low as 0.84 ng/mL with high selectivity. Bui et al. presented the protease-to-DNA converting biosensor to provide the protease activity to be transformed to generating specific DNA sequences [64]. Cy3-labeled peptide-DNAs are attached onto QD donors as the input gate. Once trypsin cleaves the peptide sequence, the donor QD emits its fluorescent signal and the DNA-Cy3 complex interacts with a tetrahedral output gate, resulting in Cy5 emission via the FRET effect. As such, in order to improve the sensitivity and simplicity of the proteolytic biosensor, the FRET phenomena are applied as a potential sensing strategy. Since the FRET-based biosensor for measuring proteolytic enzyme can sensitively change the fluorescence signal with a simple degradation reaction, it is possible to measure a small amount of proteolytic enzyme in a time-and cost-effective manner. In contrast, since FRET is only possible when the donor’s emission wavelength and the acceptor’s excitation wavelength overlap, there is a limitation to the combination of fluorescent materials. In addition, it may not be suitable for developing a multiplex biosensor that needs to measure multiple proteases at once for precise diagnosis.

On the other hand, the combination of fluorescent materials can induce a change in fluorescence property with plasmonic nanomaterials, whose surface plasmon properties can transfer absorbed optical energy into the fluorescence emission of proximal fluorescence molecules. This phenomenon is called metal-enhanced fluorescence (MEF), which substantially boosts the fluorescent signal [65]. This enhancing effect can be applied to proteolytic biosensors with similar strategies to a FRET-based analytical system. Choi et al. developed simple MEF-based proteolytic biosensors composed of DNA, peptide sequence bifunctional AuNPs, and fluorophore (fluorescein isothiocyanate; FITC) levels (Figure 1d) [66]. As the optimal distance of the MEF effect is about 8 nm between the AuNP and FITC, the quenching state is induced as the length of a single-stranded DNA (ssDNA) is 7~8 nm and the peptide sequence is smaller than the ssDNA. When the peptide sequence is degraded by caspase-3, the optimal distance of the ssDNA will show the MEF effect corresponding to the caspase-3 level. Using this biosensing system is possible for the simple (one-step proteolytic reaction) and rapid (<1 h) detection of caspase-3 as low as 10 pg/mL. This simple and sensitive detection system can also be applied to measure intracellular caspase-3. Lucas et al. exhibited MEF-based trypsin biosensors using Ag nanoparticle-modified nano-slivered 96-well plates and FITC-labeled YeBF protein [67]. The authors claimed up to 11,000× signal enhancement for fluorophores due to the effective coupling or enhancement volume region of the silver surface. This biosensing system achieved a detection limit of 1.89 ng of enzymes (2.8 mBAEE activity units). In addition, no washing steps were needed in this MEF system because of the use of the low quantum yield fluorescent label, resulting in a very low background signal of the detached fluorophore. Compared to FRET-based proteolytic biosensors, MEF-based biosensors have the advantage of excellent sensitivity due to the enhanced fluorescent signal. However, it is important to maintain an accurate distance between the donor and the acceptor (around <10 nm); hence, it is difficult to induce the MEF effect in proteolytic enzymatic detection.

### 3.2. Colorimetric Detection

**Figure 2:**
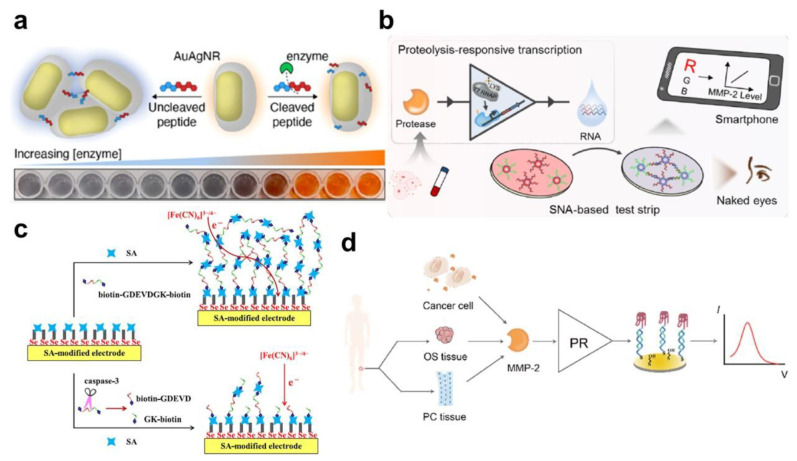
Colorimetric- and electrochemical-based biosensors for extracellular protease detection: (**a**) schematic diagram of the colorimetric biosensors using a plasmonic gold–silver core–gold nanoparticle aggregate for the measurement of trypsin activity. This figure is adapted with permission from Ref. [73] (© 2022 American Chemical Society). (**b**) Schematics of the smartphone-based colorimetric detection of MMP-2 using a modular combination of proteolysis-responsive transcription and nucleic acid-modified AuNPs. This figure is adapted with permission from Ref. [74] (© 2021 American Chemical Society). (**c**) Schematic diagram of the electrochemical protease biosensor composed of a streptavidin-modified Au electrode with a biotin–peptide–biotin complex as a caspase-3 substrate. This figure is adapted with permission from Ref. [77] (© 2021 American Chemical Society). (**d**) Label-free electrochemical detection of MMP-2 by proteolysis-triggered transcription strategy on a Au electrode. This figure is adapted with permission from Ref. [79] (© 2021 Elsevier B.V.).

Besides fluorescence-based biosensors, color change is another optical biosensor where one can observe the target proteolytic protein intuitively and easily with the naked eye. For example, lots of pregnancy tests and SARS-CoV-2 test strips have been widely used for simple diagnosis [68,69,70,71]. Change in the absorbance peak is one of the distinguished phenomena for color change. For the induction of a significant change in color or absorbance peak, AuNPs have been utilized because their absorbance property depends on their size and the degree of aggregation. Specific peptide sequences, which can be dissociated by proteolytic enzymes, can be applied to the colorimetric biosensor with AuNPs. Liu et al. developed a colorimetric biosensor for the measurement of protease activity by the growth of AuNPs [72]. Cleavage of the specific peptide against β-secretase induces the exposure of an amino-terminal Cu^2+^- and Ni^2+^-binding (ATCUN) motif. This ATCUN peptide can seize Cu^2+^ and, thus, weaken the oxidation effect of ascorbic acid, which induces the reduction of HAuCl_4_ into AuNPs. Using this strategy, β-secretase was measured as low as 0.1 nM by monitoring the color change of AuNP and ascorbic acid consumption with UV/vis spectroscopy. Creyer et al. showed a particular gold−silver core−shell nanoparticle structure for dramatic color change by the cleavage reaction between the GCGKGCG dithiol peptide and trypsin (Figure 2a) [73]. As a result, the degree of aggregation and the absorbance peak shift can be correlated with trypsin concentration with high linearity (R^2^ = 0.99). The detection limit of trypsin was 0.47 nM. Liu et al. demonstrated a colorimetric protease assay using two separate biological reactions based on proteolysis-responsive transcription (Figure 2b) [74]. MMP-2-mediated proteolysis triggers the in vitro transcription of RNAs, which induces the aggregation of DNA-functionalized AuNPs by a complimentary binding reaction. This proteolysis-responsive transcription sensor showed a sensitive detection result, as low as 3.3 pM of MMP-2. Moreover, other protease biomarkers, such as thrombin and the hepatitis C virus, could also be measured with the test strip format for smartphone analysis. Feng et al. developed a label-free peptide-AuNP biosensor for the measurement of the SARS-CoV-2 main protease (Mpro), which is one of the potential targets for the development of drugs [75]. The specific peptide sequence for the SARS-CoV-2 Mpro induces the aggregation of AuNPs by electrostatic interaction. Once the Mpro degrades the peptide, the AuNPs are dissociated and the absorbance peak is blue-shifted, resulting in a clear color change. Moreover, the electrode-modified peptide can be aggregated with the AuNPs, and the proteolytic event can induce a change in the electrochemical signal. In the colorimetric and electrochemical sensing systems, the detection limits were 10 and 0.1 pM, respectively. Ling et al. proposed a nanocellulose-based colorimetric biosensor for the facile detection of human neutrophil elastase [76]. Due to its crystallinity, high surface area, and biocompatible properties, nanocellulose can be utilized as an efficient sensing transducer. Through a deep eutectic solvent treatment, cotton cellulose nanocrystals are formed, and they modify specific peptides for the human neutrophil elastase. The sensitivity of this colorimetric sensor is around 0.005 U/mL. The authors claimed that it could provide a sensitive and convenient sensor platform applicable for point-of-care protease detection.

### 3.3. Electrochemical Detection

Besides the optical biosensing approaches for protease biomarker detection, electrochemical measurement has been developed using specific peptides and the corresponding proteases. Xia et al. proposed a novel protease biosensor by altering a homogeneous assay into a surface-bound electrochemical analysis (Figure 2c) [77]. A streptavidin-covered electrode functionalizes the biotin-specific peptide–biotin complex for streptavidin–biotin coupling chemistry. The repeated formation of streptavidin−biotin−GDEVDGK−biotin aggregates provides an insulating layer, thus reducing the electron transfer of ferricyanide. If the peptide sequence is cleaved by caspase-3, the resulting products are distributed into a bulk solution and electron transfer is increased. Therefore, the amplification of the electrochemical signal is achieved with the simple principle of substrate-induced streptavidin assembly. Zhang et al. developed a thiol-sensitive electrochemical probe for the measurement of the SARS-CoV-2 main protease, targeted by a short probe mimicking its substrate [78]. In the probe–protein interaction, a specific fluorescent molecule interacts with the free thiol groups on the target protein, producing a fluorescence and electrochemical signal proportional to the target concentration. The LOD of the SARS-CoV-2 main protease was 1 pM, whereas the clinically required LOD was around 182 pM. The authors claimed that this analytical method could measure the virus protease in clinical SARS-CoV-2-infected patient samples in a simple one-step reaction and in a reagent-less fashion. Shi et al. revealed a label-free electrochemical biosensor for the sensitive detection of MMP-2, with signal amplification using a proteolysis-triggered transcription method (Figure 2d) [79]. The authors utilized RNA polymerase to transduce and amplify the proteolysis reaction by MMP-2 into multiple RNA productions that can be bound by the complementary DNA probes modified on the Au electrode. By integrating the G4/hemin complex, this RNA polymerase-assisted electrochemical biosensor facilitates the highly sensitive detection of MMP-2, with a wide dynamic range from 10 fM to 1.0 nM and 7.1 fM LOD value. Eissa et al. developed a specific peptide sequence-functionalized magnetic bead biosensor for the detection of *Staphylococcus aureus* using dual colorimetric and electrochemical analyses [80]. The peptide-magnetic bead complex is immobilized on the screen printed Au electrode. The protease released from *Staphylococcus aureus* degrades the specific peptide, and the color of the Au electrode changes due to the dissociation of the magnetic bead. In addition, the protease can be detected by monitoring the change in the peak current of square wave voltammetry within 1 min. The LOD value of this electrochemical assay was 3 CFU ml^−1^, and it was tested with a spiked milk and water sample.

### 3.4. Others

Because the peptide-cleavage reaction can change various output signals, including fluorescence and electrochemical readouts, other simple and sensitive biosensing approaches have also been researched. Among them, bioluminescence resonance energy transfer (BRET)-based biosensors are similar to the sensing mechanism of FRET. Weihs et al. developed a red-shifted BRET biosensor for the measurement of plasmin activity [81]. Conventional BRET-based proteolytic biosensors use the blue-shifted BRET system, which suffers from background signals due to light absorption and scattering in plasma samples. To overcome this limitation, the authors applied a red-shifted RLuc8.6 luciferase instead of Renilla luciferase Rluc2. As a result, the proposed biosensor exhibited up to a 5-fold increase in sensitivity for plasma samples as low as 11.90 nM within 10 min. In addition, they also presented the real-time-on-chip detection of thrombin activity by integrating the BRET phenomenon [82]. The real-time-on-chip is composed of a compact microreactor and a reusable glass chip with a mixer, an incubation channel, and a detection chamber. This compact chip platform provides a minimum of handling steps, which can reduce the handling error for the precise measurement of thrombin. This sensitive chip can detect thrombin as low as 38 μU/μL in human serum within only 5 min, which is 90% faster than conventional assays.

Surface-enhanced Raman spectroscopy (SERS) could also be a potential sensitive biosensing method for protease detection. Due to the proportional signal intensity to the distance between the Raman dye and SERS-active substrates, the peptide-cleavage event can induce a dramatic change in SERS signals. Choi et al. revealed a SERS-active platform consisting of an Ag-coated hollow polypyrrole (hPPy) nanohorn with a specific peptide sequence [83]. To maximize the SERS signal, Ag is electrodeposited on both sides of the hPPy nanohorn structure. Using this core/double-shell nanohorn substrate, the Raman signal of the peptide-functionalized AuNPs is significantly improved, and caspase-3 can be sensitively measured, with a wide detection range from 10 pg/mL to 10 μg/mL. Wei et al. developed O-GlcNAc transferase (OGT) biosensors by integrating SERS tags and a magnetic bead complex [84]. The SERS tags and magnetic bead complex are linked to the two ends of the peptide by thiolated and biotin residues. In the presence of OGT, peptide glycosylation protects the peptide from the cleavage reaction by proteinase K, resulting in more SERS signals from the left Raman tags on the surface of magnetic beads. This system can sensitively detect OGT as low as 0.1 nM, ranging from 10^−10^ to 10^−7^ M. Besides optical analytical methods, a giant magnetoresistive spin-valve (GMR SV) sensor has been developed for the detection of cysteine protease [85]. Magnetic nanoparticles are immobilized on the surface of GMR SV using a cysteine-protease-specific peptide. This magnetic field measurement approach can detect papain, one of the cysteine proteases, as low as 4 nM in only 3.5 min.

## 4. Intracellular Detection of Protease for In Situ Monitoring

### 4.1. In Vitro Proteolytic Analysis at a Cellular Level

As described in Section 3, since extracellularly released proteases can be biomarkers of specific diseases, the development of sensitive and selective biosensors is necessary for the accurate and early diagnosis of several diseases. In addition to measuring extracellular protease in vitro, it is also possible to measure intracellular proteases quantitatively and qualitatively. As illustrated above, as measuring proteases does not involve complex reactions such as antigen–antibody reactions, intracellular proteases can be measured in a simple manner and in real-time. Because intracellular proteases, similar to extracellular proteases, are important biomarkers for determining the state of cells, many researchers have studied intracellular protease biosensors using specific peptide degradation.

For the detection of intracellular proteases, fluorescence-based analytical methods have been mainly utilized using the distance-dependent FRET phenomenon. In particular, there have been virus-infected cells that are enabled to measure virus-related proteases in real-time to diagnose viral infection. Guerruiro et al. exhibited a genetically-engineered turn-on fluorescent biosensor containing a cyclized green fluorescent protein (GFP)-conjugated cVisensor with a specific peptide sequence for adenoviral protease detection [86]. Structural distortion of circular permuted superfolder-GFP is cyclized by *Nostoc punctiforme* DnaE. This in situ live cell biosensor works to restore the deformation by the peptide-cleavage reaction and emits the fluorescent signal of GFP. This label-free sensing system can detect adenovirus 2 days post-infection, faster than conventional plaque-forming assays that require 14 days. Dey-Rao et al. developed a live cell-based fluorescence biosensor to evaluate the intracellular function of the SARS-CoV-2 main protease and its inhibitor [87]. The authors designed it to express a red fluorescence protein (RFP) biosensor unless the SARS-CoV-2 main protease cleaves the peptide and to facilitate the loss of the fluorescence emission of RFP. Inhibition of the main protease function provides the synthesis of working RFP, resulting in the recovery of fluorescence. In addition, GC376, which is the pan-coronavirus’s main protease inhibitor, shows effective inhibition of the intracellular CoV2 main protease. The authors claim that this intracellular sensing system can offer an extremely efficient high-throughput screening system for the SARS-CoV-2 main protease. Guerreiro et al. developed GFP-based switch-on split fluorescent biosensors to examine viral infection [88]. Through the protein distortion, which is composed of GFP11 embedment distortion (SNR 6.0) or GFP11 cyclization (SNR 3.5), GFP is split and cannot be assembled for the fluorescent emission. Once adenovirus- and lentivirus-promoted proteolysis cleaves the peptide sequence, this intracellular biosensor shows the fluorescent signal in live cells after 24 h post-infection with a high signal-to-noise ratio of up to 97. Gerber et al. revealed a live cell-based luminescent biosensor for the verification of SARS-CoV-2 infection using recombinant SARS-CoV-2 proteases [89]. This luminescent-based assay applies the cleavage of specific peptide linkers, which are degraded by the main protease of SARS-CoV-2, allowing viral infection to be measured within 24 h in the multi-well plate format for high-throughput analysis. The authors claim that the developed luminescent SARS-CoV-2 reporter live-cell-based biosensor can demonstrate the comparative quantitation of the SARS-CoV-2 virus and the titration of neutralizing antibodies.

**Figure 3:**
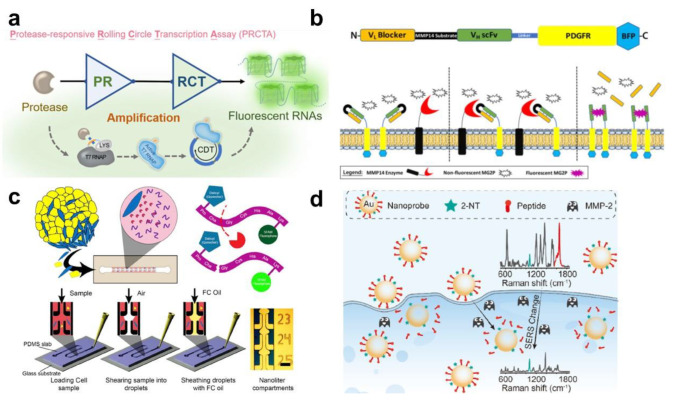
In vitro proteolytic analyzing biosensors: (**a**) Schematic diagram of the proteolysis-responsive rolling circle transcription assay (PRCTA) by integrating protease-responsive RNA polymerases and rolling circle transcription. This figure is adapted with permission from Ref. [90] (© 2020 American Chemical Society). (**b**) Plasmid-based cell-surface fluorescent biosensor for measurement of the location and activity of MMP-14. This figure is adapted with permission from Ref. [91] (© 2018 Springer). (**c**) Microfluidic capillary biosensor for quantifying secreted protease activity at the single cell level. This figure is adapted with permission from Ref. [95] (© 2020 Elsevier B.V.). (**d**) SERS imaging of intracellular MMP-2 activity with the ratiometric SERS Nanoprobe. This figure is adapted with permission from Ref. [96] (© 2020 Elsevier B.V.).

Besides virus-related protease detection, other intracellular proteases have also been measured with specific peptide-cleavage reactions in live cells. Luo et al. showed the highly sensitive and precise detection of MMP-2 activity using a rolling circle transcription assay-integrated proteolytic reaction (Figure 3a) [90]. The proteolytic reaction induces the activation of the bacteriophage T7 RNA polymerase (T7 RNAP) and produces long RNA chains, including tandem G-quadruplexes (G4s). As a result, the activity of MMP-2 can be transduced into multiple fluorescent signals from G4s RNAs. For verification of the sensing principle for cell imaging, six distinct cell lines with different MMP-2 expression levels were employed, and they indicated different signals for the MMP-2 expression levels. Braun et al. exhibited a cell-surface-displayable biosensor for MMP-14 detection (Figure 3b) [91]. The cell-surface biosensor consisted of two scFv domains; one was blocked against MG2P fluorescent dye, and another was scFv, a connected specific MMP-14 cleavable sequence. Once the cell-surface MMP-14 degrades the peptide bridge, the blocker is dissociated, and the fluorogen (MG2P) is bound to the scFv domains. Subsequently, the MMP14–fluorogen (MG2P) complex is activated, and the fluorescence signal is generated. This switch-on cell-surface biosensor was enabled to measure the activity of MMP14, as well as location and temporal dynamics. Xu et al. developed a multi-color fluorescent polydopamine nanobiosensor for the multiple sensing of cancer-related proteases in living cells, such as urokinase-type plasminogen activator (uPA), cathepsin B (CTB), matrix metalloproteinase-2, and matrix metalloproteinase-7 (MMP-7) [92]. The polydopamine nanoprobe is functionalized by the four different peptide sequences with different fluorescent dyes. Due to the quenching effect of the polydopamine nanoprobe, the four fluorescent dyes cannot emit a signal. If the polydopamine nanoprobe is intracellularly delivered into the cancer-related cells, the intracellular protease can particularly cleave each peptide, allowing the recovery of the fluorescent signals. Due to the multiplexed intracellular detection, this sensing system can evaluate the stages of tumor progression and provide early and multiple diagnoses of cancers. Peyressatre et al. exhibited a cyclin-dependent kinase 5 (CDK5) kinase activity biosensor for the diagnosis of neurodegenerative pathologies, including glioblastoma and neuroblastoma [93]. The authors developed a CDK5-specific fluorescent peptide biosensor through the selection of peptide sequences from the CDK5 substrate. The quantification of CDK5 kinase activity can be measured on treatment with ATP-competitive inhibitors in both cell extracts and the living cell environment by time-lapse fluorescence microscopy. These kinds of intracellular biosensing systems can facilitate the evaluation of the functional status of intracellular proteases in several cancers and neurodegenerative pathologies by real-time monitoring. The intracellular biosensing method can also be applied to floating cells, such as immune cells, for monitoring endogenous metabolic processes during the immune response. Sun et al. developed a genetically programmed fluorescent itaconate biosensor (BioITA) for the immediate monitoring of itaconate dynamics, which is an anti-inflammatory program of the innate immune response in living macrophages [94]. The authors succeeded in monitoring the itaconate progress by stimulating lipopolysaccharides in living macrophages. In addition, through the BioITA, the response and changing itaconate level in activated macrophages could be observed by the injection of an adeno-associated virus (AAV) with spatiotemporal resolution. Hassanzadeh-Barforoushi et al. utilized microfluidic channels for the measurement of released proteases from single cells using capillary force-assisted separation (Figure 3c) [95]. For the isolation and easy and reliable capture of a small number of cells (~500 single cells), the capillary-stoppage method was applied to the microfluidic channel by shearing with air and sheathing with FC-40 oil. Key parameters, including single-cell encapsulation efficiency (38.8%) and droplet volume evaporation rate (10%) in 48 h, were achieved in the microfluidic platform, and the manipulation of droplet composition through controlled gradient generation were studied. Based on the separation of nanoliters at the single-cell level, the authors employed a simple FRET-based biosensor for the monitoring of secreted MMP-2 activity. The authors claim that this microfluidic platform offers a rapid, simple, and single-cell-level detection method without engineering expertise. Zhong et al. developed an in situ ratiometric SERS nanoprobe for the intracellular imaging of proteases (MMP-2) in different cancer cells for sensitive detection (Figure 3d) [96]. Due to the advantages of the SERS analytical method, such as high sensitivity, resistance to photobleaching and quenching, and trivial autofluorescence, SERS-based visualization using the 2-naphthalenethiol (NT)-labeled Au nanoprobe was applied to measure MMP-2 activity in single living cancer cells. Upon the internalization of the Au nanoprobe into cancer cells, the proteolytic cleavage of the peptide sequence resulted in the dissociation of the 2-NT molecules, and the Raman signal was decreased. Live cell imaging results showed that the MMP-responsive nanoprobe differentiated normal breast cells from breast cancer cells and also differentiated two different breast cancer cell lines with different malignant properties. Cheng et al. developed a selective in vivo imaging and inhibition system against SARS-CoV-2 infection. This amphiphilic assembly system consists of aggregation-induced emission (AIEgen), self-assembly, spacer, and main protease-responsive and cell-penetrating peptide domains. The degradation of this amphiphilic assembly by the main protease from SARS-CoV-2 induces aggregation with increased fluorescence and mitochondrial interference of the infected cells. As a result, this system can perform the selective bioimaging and treatment of SARS-CoV-2-infected cells [97].

### 4.2. In Vivo Proteolytic Analysis for Bioimaging

**Figure 4:**
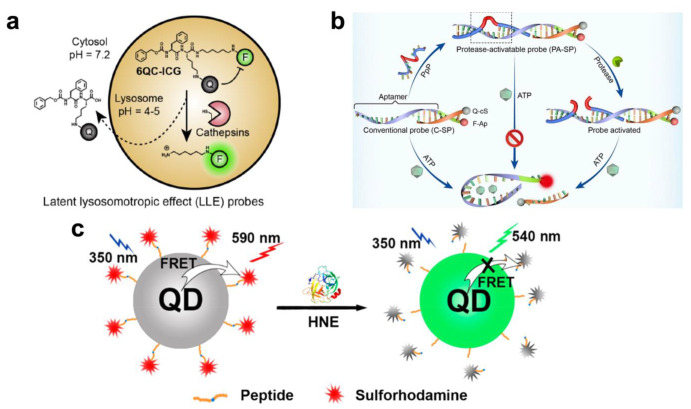
In vivo proteolytic analysis of intracellular proteases for bioimaging: (**a**) Schematics of the fluorescence-guided detection of otitis media (OM) using a protease-cleavable biosensor. This figure is adapted with permission from Ref. [98] (© 2020 American Chemical Society). (**b**) PNA-assisted DNA aptamer sensor with peptide for cathepsin B-activatable ATP detection. This figure is adapted with permission from Ref. [100] (© 2021 Wiley-VCH). (**c**) FRET-based neutrophil elastase biosensor for in vitro detection and in vivo imaging using the peptide substrate, QDs, and organic dyes. This figure is adapted with permission from Ref. [102] (© 2020 American Chemical Society).

Proteolytic cleavage-based biosensing strategies can be applied to in vivo imaging using optical probes such as fluorescent molecules. Yim et al. developed ear inflammatory biosensors by measuring the short-wave infrared (SWIR) otoscope (Figure 4a) [98]. Cysteine cathepsin proteases, which are upregulated in the inflamed state by immune cells, are released from the otitis media (middle ear infection). The fluorescent probe is linked to the quencher (6QC-ICG) through the cleavable peptide, and this probe is emitted into the lysosome by the latent lysosomotropic effect in acute otitis media. As a result, middle ear infection shows a clear fluorescent emission with a 2.0 signal-to-background ratio. Moore et al. demonstrated a novel detection method for in vivo periodontal inflammation by measuring gingipain proteases released by *P. gingivalis* [99]. For the transduction of a specific peptide cleavage into the signal, the activatable photoacoustic and fluorescent molecular probe, which consisted of a dye-conjugated peptide, [Cy5.5]_2_[APRIK], was utilized as an imaging agent for gingipain proteases. The sensitivity of this in vivo biosensor showed 5-fold photoacoustic and >100-fold fluorescence enhancement, with 1.1 nM and 4.4 × 10^−4^ CFU/mL LOD. In addition, the photoacoustic imaging of the gingipain protease probe was demonstrated in the subgingival pocket of porcine mandibles and the murine brain due to the check on the biological relationship between gingipain and Alzheimer’s disease. Xiang et al. exhibited a DNA/peptide/PNA triblock copolymer for in vitro ATP imaging and in vivo tumor imaging (Figure 4b) [100]. The sensing system is composed of a rationally designed peptide nucleic acid (PNA)–peptide–PNA triblock copolymer, which can be divided by tumor-overexpressed cathepsin B. The cathepsin B-activatable probe is in double-stranded form, which is the Cy5-labeled DNA aptamer strand and the quencher (BHQ2)-labeled short complementary DNA strand. The cathepsin B-induced activation of the probe enables tumor cell-specific molecular imaging by the folding of the aptamer with binding ATP, and the fluorescence signal is recovered. This ATP sensing-based fluorescence imaging probe can selectively measure the cathepsin B protease in tumor cells both in vitro and in vivo. Kang et al. developed a chronic wound evaluation platform by integrating a fluorophore–peptide quencher into a maleimide-functionalized polyethylene glycol–diacrylate (PEG-DA) hydrogel [101]. Using the in vivo fluorescence imaging method, a high level of MMP-2 and MMP-9 was clearly demonstrated on the chronic wound site. The authors claim that this simple protease sensor can be applied to indicate the severity of the wound to the surgical doctor and other medical staff by providing the patch format of a fluorescence sensor. Liu et al. reported a noninvasive diagnostic platform of common pulmonary diseases by measuring the activation of human neutrophil elastase (Figure 4c) [102]. This imaging probe consists of a QD–human neutrophil elastase-specific peptide substrate fluorescent dye for the induction of the FRET effect. This system showed both the in vitro and in vivo detection of human neutrophil elastase, with 7.15 pM in aqueous solution, and clear imaging in lung cancer and acute lung injury mouse models. Using this sensor could differentiate pulmonary patients from the healthy with the minimization of environmental interference of the fluorescence.

## 5. Outlook and Conclusions

In this review, proteolytic enzymes that can be used as biomarkers for diagnosing various diseases have been briefly demonstrated, with the latest research trends showing the integration of functional nanomaterials. As mentioned above, proteolytic enzymatic biosensors have various advantages over immunoassays represented by ELISA. First, the measurement process of proteolytic biosensors is simpler than ELISA. Compared to multiple antigen–antibody reactions (two or more separated reactions), proteolytic enzymes can be measured using only a peptide degradation reaction. In addition, it is cost-effective and time-saving as the complexity of the measurement can be reduced. Unlike ELISA, one of the advantages is that the additional labeling step of the signaling molecule is not required after the biological reaction (i.e., antigen–antibody reaction) of the target biomarker. Finally, false-negative and false-positive signals caused by antibody instability can also be reduced. However, while most proteins have their specific antibodies, there is a limitation that only proteolytic biomarkers with specific enzymatic properties can be measured. This is a major disadvantage that reduces the versatility of the sensor.

Recently, CRISPR-based DNA and RNA biosensors have been actively studied for sensitive and simple detection. These analytical methods are based on random single nucleic acid degradation reactions if the target DNA/RNA lets the CRISPR complex activate, similar to the strategies of proteolytic enzymatic biosensors. Since the decomposition reactions of peptides and nucleic acids are irreversible, these kinds of sensors cannot be reused. Paradoxically, the non-reusability of the biosensor could be an advantage, namely, as a disposable sensor, because it can improve accuracy by reducing errors due to the reduction of biological reactions due to the regeneration of sensors. In addition, a system for measuring multiple biomarkers is essential for the accurate and early diagnosis of diseases. From this point of view, the proteolytic sensor can easily apply a multiplex system because the peptides that each proteolytic enzyme degrades are different. Furthermore, integration with the CRISPR sensor enables the simultaneous measurement of various types of biomarkers, such as DNA, RNA, and other proteins, on one platform. This will provide not only the accurate diagnosis of the disease mentioned above but also detailed disease information, for example, the progress of the disease and the prognosis after treatment.

Studies measuring the activity of proteolytic enzymes can be applied to various biomedical fields besides in vitro diagnosis. For example, it can be used for real-time in vivo imaging using intracellular and extracellular proteolytic enzymes. In addition, when proteolytic enzymes are used with therapeutic drugs and nanoparticles, they can be used for the development of nanotheragnosis, which can be treated and diagnosed at the same time. For instance, since the MMP family are typically highly expressed in various cancers, it will be possible to develop nanotherapeutics with high potential by inducing drug release through peptide degradation events while detecting them in the body. In this way, it is believed that in combination with various nanotechnology, it will be possible to secure a platform technology that can simultaneously perform various functions while improving measurement sensitivity. In addition, proteolytic biosensors could be applied to toxicological and pharmacological screening systems. For example, sentrin-specific protease 1, which is related to many diseases, including cancers and cardiovascular diseases, is measured for the identification of anti-cancer agents that have toxic effects [103]. Another example is the SARS-CoV-2 main protease inhibitors screened using a proteolytic-based biochemical high-throughput screening (HTS) system. These screening platforms are feasible for simple and sensitive tests for drug screening and pharmacological applications [104]. Proteolytic enzymatic biosensors are expected to be used more actively in the field of early diagnosis of diseases in the future, and it is expected to be widely used for basic biological research, in vitro model systems with sensitive analysis platforms at the cell level, and other biomedical fields such as drug delivery and in vivo imaging.

## Data Availability

Not applicable.
